# Breast cancer, viruses, and human leukocyte antigen (HLA)

**DOI:** 10.1038/s41598-024-65707-9

**Published:** 2024-07-13

**Authors:** Lisa M. James, Apostolos P. Georgopoulos

**Affiliations:** 1 The HLA Research Group, Department of Veterans Affairs Health Care System, Brain Sciences Center, Minneapolis VAMC, One Veterans Drive, Minneapolis, MN 55417 USA; 2 Department of Neuroscience, University of Minnesota Medical School, Minneapolis, MN USA; 3 Department of Psychiatry, University of Minnesota Medical School, Minneapolis, MN USA; 4 Institute for Health Informatics, University of Minnesota Medical School, Minneapolis, MN USA

**Keywords:** Breast cancer, Viruses, Human leukocyte antigen (HLA), Immunogenicity, Cancer, Computational biology and bioinformatics, Immunology, Diseases, Medical research, Oncology

## Abstract

Several viruses have been implicated in breast cancer, including human herpes virus 4 (HHV4), human herpes virus 5 (HHV5), human papilloma virus (HPV), human JC polyoma virus (JCV), human endogenous retrovirus group K (HERVK), bovine leukemia virus (BLV) and mouse mammary tumor virus (MMTV). Human leukocyte antigen (HLA) is involved in virus elimination and has been shown to influence breast cancer protection/susceptibility. Here we investigated the hypothesis that the contribution of a virus to development of breast cancer would depend on the presence of the virus, which, in turn, would be inversely related to the success of its elimination. For that purpose, we estimated in silico predicted binding affinities (PBA) of proteins of the 7 viruses above to 127 common HLA alleles (69 Class I [HLA-I] and 58 Class II HLA-II]) and investigated the association of these binding affinities to the breast cancer—HLA (BC-HLA) immunogenetic profile of the same alleles. Using hierarchical tree clustering, we found that, for HLA-I, viruses BLV, JCV and MMTV were grouped with the BC-HLA, whereas, for HLA-II, viruses BLV, HERVK, HPV, JCV, and MMTV were grouped with BC-HLA. Finally, for both HLA classes, the average PBAs of the viruses grouped with the BC-HLA profile were significantly lower than those of the other, non BC-HLA associated viruses. Assuming that low PBAs are likely associated with slower viral elimination, these findings support the hypothesis that a defective/slower elimination and, hence, longer persistence and inefficient/delayed production of antibodies against them underlies the observed association of the low-PBA group with breast cancer.

## Introduction

Breast cancer is the most common cancer affecting women worldwide^1^. Several risk factors have been identified, including family history of breast cancer, dense breast tissue, female reproductive factors, alcohol or tobacco use, body mass index, and genetics (e.g. BRCA gene)^2,3^; however, nearly half of breast cancers develop in women in the absence of these risk factors^3^, suggesting that additional factors likely contribute to breast cancer risk.

The role of viruses in breast cancers is increasingly recognized and is likely underestimated^4^. As documented elsewhere, human herpes viruses (HHV) including Epstein Barr virus (EBV, HHV4) and cytomegalovirus (CMV, HHV5), mouse mammary tumor virus (MMTV), high risk human papilloma virus (HPVs), bovine leukemia virus (BLV), human polyomavirus JC virus (JCV), and human endogenous retrovirus K (HERVK) have been implicated in human breast cancer^4–9^. Several viruses (e.g., MMTV, HPV, EVB, and BLV) have been identified and shown to co-exist in human breast cancer cells^10–12^, and in benign breast biopsies 1–11 years before developing cancer^11^. They have also been identified in normal breast tissue samples and in milk of normal lactating women, albeit to a lesser extent^10,11^. Indeed, viruses linked to human cancers are ubiquitous yet only a small proportion of infected individuals develop cancer, one of many reasons that have made it challenging to identify causal relations between viruses and cancer^13^. One factor that may moderate the association between viruses and breast cancer is variation in host immunogenetics related to human leukocyte antigen (HLA).

HLA genes, located on chromosome 6, code for two main classes of cell-surface proteins involved in the immune response to foreign antigens including viruses and cancer neoantigens^14,15^. HLA-I molecules of the classical genes A, B and C are expressed on all nucleated cells, bind and present small peptides (8–10 amino acid residues^16^) from proteolytically degraded foreign antigens to CD8 + cytotoxic T cells, signaling cell destruction. HLA-II molecules of the DPB1, DQB1 and DRB1 genes are expressed on lymphocytes and professional antigen presenting cells, present larger peptides (12–22 amino acid residues^17^) derived from endocytosed exogenous antigens to CD4 + T cells, facilitating antibody production and adaptive immunity. Each individual carries two of each HLA gene, for a total of 12 classical HLA alleles. The HLA region is the most highly polymorphic region of the human genome^18^, with most of the variation existing in the binding groove. This variation amounts to tremendous individual variability in the ability to bind and eliminate viruses and other foreign antigens. Specific HLA alleles have been associated with breast cancer protection or susceptibility^19–28^. This association is captured in the breast cancer—HLA immunogenetic profile which contains the correlations between the prevalence of breast cancer and HLA allele frequency^19^. Given the documented involvement of several viruses in breast cancer, discussed above, we investigated, in this study, the possible viral elimination by the HLA system, as a mechanism of preventing the oncogenic effect of those viruses. More specifically, we focused on 7 viruses that have been found in breast cancer tissue (HHV4, HHV5, HPV, JCV, MMTV, BLV, HERVK) and estimated in silico their binding affinity with respect to 69 common HLA-I alleles of the 3 classical genes (A, B, C) and 58 common HLA-II alleles of the 3 classical genes (DPB1, DQB1, DRB1). Since binding affinity is a critical initial step in foreign antigen elimination, it is reasonable to assume that high binding affinity would be more effective in virus elimination, and vice versa for low binding affinity. Thus the objectives of this study were (a) to estimate in silico the predicted binding affinity of specific viruses with respect to specific alleles using the Immune Epitope Database (IEDB) NetMHCpan (ver. 4.1) tool^29,30^, (b) to identify those viruses whose binding affinities were associated with the breast cancer—HLA immunogenetic profile, and (c) to test the hypothesis that the predicted binding affinity of this set of viruses is lower than that of the viruses unassociated with the breast cancer—HLA profile.

## Results

### General

Predicted Binding Affinity (PBA) scores varied substantially among virus proteins (Table 1) and HLA-I alleles (Table 2) and HLA-II (Table 3). All HLA-I PBA values (N = 69 alleles × 7 viruses = 483) were positive (indicating high affinity lowest percentile rank (LPR) < 1, PBA > 0), whereas for HLA-II, 12.8% (52 out of N = 58 alleles × 7 viruses = 406) were negative (indicating low affinity LPR > 1, PBA < 0).

**Table 1:** Viral proteins used.

	Virus	Protein description	UniprotKB ID	N (AA)
HHV4	Human Herpes Virus 4	Envelope glycoprotein B	P03188	857
HHV5	Human Herpes Virus 4	Envelope glycoprotein B	P06473	906
HPV	Human Papilloma Virus	Major capsid protein L1	Q81007	494
JCV	JC polyomavirus (JCV)	Major capsid protein VP1	P03089	354
HERVK	Human Endogenous Retrovirus group K	10 Pol protein	P10266	1014
BLV	Bovine Leukemia Virus	Envelope glycoprotein	P51519	515
MMTV	Mouse Mammary Tumor Virus	Envelope glycoprotein gp70	P03374	688

**Table 2:** Predicted binding affinities (PBA, see “Methods”) and Breast Cancer—HLA P/S scores^17^ for all 69 HLA-I alleles and 7 viruses studied.

Index	Allele	Gene	HHV4	HHV5	HPV	JCV	HERVK	BLV	MMTV	BC-HLA P/S score
1	A*01:01	A	4.605	3.507	2.659	1.050	4.605	2.303	3.507	0.068
2	A*02:01	A	3.912	2.996	2.526	2.207	3.507	2.813	2.408	0.086
3	A*02:05	A	3.507	3.912	3.912	2.303	3.912	3.219	3.507	− 0.071
4	A*03:01	A	2.526	3.912	2.996	2.813	3.912	0.446	1.966	0.042
5	A*11:01	A	4.605	3.912	3.912	1.772	3.507	2.120	3.219	− 0.034
6	A*23:01	A	2.813	3.507	2.813	1.897	2.303	3.912	2.813	− 0.519
7	A*24:02	A	3.507	3.507	2.996	1.897	2.659	3.507	3.219	− 0.169
8	A*25:01	A	3.507	3.912	3.912	4.605	3.507	3.912	2.040	− 0.188
9	A*26:01	A	3.507	3.912	3.912	4.605	3.912	3.912	2.207	− 0.326
10	A*29:01	A	3.219	3.912	3.912	3.219	4.605	3.507	3.219	− 0.043
11	A*29:02	A	3.219	3.912	3.912	3.219	4.605	3.507	3.219	− 0.210
12	A*30:01	A	3.912	3.912	2.659	3.912	3.912	3.912	3.912	− 0.080
13	A*30:02	A	3.912	3.912	3.507	3.219	3.912	2.813	3.507	− 0.114
14	A*31:01	A	3.507	2.813	3.219	1.514	3.507	3.219	3.912	− 0.015
15	A*32:01	A	3.507	4.605	2.996	2.813	3.507	2.996	2.526	− 0.054
16	A*33:01	A	2.408	3.912	4.605	3.507	3.507	2.040	3.912	− 0.118
17	A*33:03	A	2.040	3.507	4.605	2.996	3.912	2.408	2.526	− 0.058
18	A*36:01	A	2.996	3.912	3.219	0.844	3.912	2.526	3.507	− 0.196
19	A*68:01	A	3.507	3.912	3.912	1.427	3.912	2.408	3.507	0.318
20	A*68:02	A	2.996	3.507	3.912	2.408	2.813	2.408	3.507	− 0.669
21	B*07:02	B	3.507	2.120	2.120	3.507	2.408	3.912	3.219	− 0.007
22	B*08:01	B	3.912	3.219	3.219	1.079	3.507	3.912	2.813	0.283
23	B*13:02	B	3.912	3.912	1.833	1.833	3.912	3.912	2.813	− 0.255
24	B*14:01	B	3.912	3.507	3.219	1.347	3.912	3.507	3.507	− 0.262
25	B*14:02	B	3.912	3.507	3.219	1.347	3.912	3.507	3.507	− 0.023
26	B*15:01	B	4.605	3.912	3.507	3.219	3.912	3.507	3.219	0.073
27	B*15:17	B	2.659	3.912	1.609	3.507	3.912	2.526	2.996	0.341
28	B*15:18	B	4.605	3.219	3.507	2.408	4.605	2.996	2.996	0.151
29	B*18:01	B	4.605	4.605	4.605	2.526	2.813	2.996	1.715	− 0.100
30	B*27:02	B	2.408	2.408	1.966	1.833	2.526	2.207	3.219	− 0.007
31	B*27:05	B	2.040	2.996	1.715	2.303	3.507	2.813	4.605	− 0.071
32	B*35:01	B	3.219	4.605	3.219	2.526	3.219	2.813	4.605	0.393
33	B*35:02	B	3.507	2.996	2.996	3.507	3.507	3.507	3.507	− 0.275
34	B*35:03	B	3.507	2.526	2.996	3.507	3.507	3.507	3.507	− 0.064
35	B*35:08	B	3.912	3.912	3.912	2.120	3.507	2.813	3.912	0.101
36	B*37:01	B	3.219	3.507	3.912	3.912	3.507	3.507	3.507	0.334
37	B*38:01	B	3.912	3.912	3.912	0.844	4.605	3.912	2.040	− 0.275
38	B*39:01	B	3.912	3.912	3.912	1.833	4.605	3.507	2.526	0.148
39	B*39:06	B	3.219	3.219	3.912	1.238	3.912	3.912	3.912	0.120
40	B*40:01	B	3.507	2.996	2.303	3.219	3.219	4.605	2.120	0.205
41	B*40:02	B	2.659	2.813	3.507	2.659	2.040	3.912	2.996	− 0.143
42	B*41:01	B	3.219	2.526	3.912	2.408	3.219	3.219	3.507	− 0.088
43	B*41:02	B	2.659	2.996	4.605	2.659	2.659	3.507	3.912	− 0.242
44	B*44:02	B	3.507	3.507	2.813	0.562	4.605	3.219	2.526	− 0.417
45	B*44:03	B	3.912	3.912	3.912	0.562	4.605	2.996	2.303	− 0.316
46	B*44:05	B	2.659	3.912	3.507	1.561	3.912	3.912	3.507	0.151
47	B*45:01	B	3.219	2.659	3.912	2.303	3.219	2.659	3.219	− 0.045
48	B*47:01	B	3.219	3.507	3.219	2.526	2.207	3.507	3.219	− 0.226
49	B*49:01	B	2.659	4.605	2.303	1.661	3.912	3.912	3.912	− 0.081
50	B*50:01	B	2.996	2.659	3.507	2.659	2.996	2.040	3.507	− 0.356
51	B*51:01	B	2.408	3.912	3.912	3.507	3.912	2.120	3.507	0.012
52	B*52:01	B	2.303	3.507	1.966	3.912	4.605	4.605	3.912	− 0.240
53	B*55:01	B	3.912	3.912	2.120	2.120	3.219	2.526	3.219	0.324
54	B*56:01	B	3.507	3.912	2.207	2.040	2.813	2.303	3.912	− 0.007
55	B*57:01	B	2.303	2.207	0.916	3.507	2.659	3.507	3.912	− 0.105
56	B*58:01	B	2.813	2.526	1.470	3.507	2.659	3.912	3.912	0.034
57	C*01:02	C	3.912	3.219	3.912	2.120	3.912	3.912	3.912	− 0.082
58	C*03:03	C	2.813	4.605	3.219	2.813	3.912	3.507	3.912	0.250
59	C*04:01	C	3.912	2.996	2.996	2.040	2.996	1.833	2.996	− 0.171
60	C*05:01	C	4.605	3.912	2.996	1.139	3.507	3.507	2.996	− 0.227
61	C*06:02	C	3.219	3.219	3.912	3.912	3.507	2.996	2.659	0.066
62	C*07:01	C	3.912	3.912	3.912	3.912	3.507	2.996	2.659	0.364
63	C*07:02	C	3.912	4.605	4.605	3.912	3.219	4.605	2.813	0.383
64	C*07:04	C	3.912	3.912	3.507	2.408	3.912	3.219	3.219	− 0.131
65	C*12:02	C	2.408	4.605	3.507	2.120	3.912	3.219	3.507	− 0.049
66	C*12:03	C	2.207	4.605	2.996	1.609	3.912	2.526	3.912	− 0.050
67	C*14:02	C	3.912	3.912	4.605	3.507	3.507	3.912	3.912	− 0.006
68	C*15:02	C	3.507	3.912	3.912	2.996	3.912	3.219	3.912	− 0.191
69	C*16:01	C	2.659	4.605	2.207	2.813	3.912	3.219	3.912	− 0.425

**Table 3:** Predicted binding affinities (PBA, see “Methods”) and Breast Cancer—HLA P/S scores^17^ for all 58 HLA-II alleles and 7 viruses studied.

Index	Allele	Gene	HHV4	HHV5	HPV	JCV	HERVK	BLV	MMTV	BC-HLA P/S score
1	DPB1*01:01	DPB1	1.609	1.386	0.562	0.942	1.347	0.386	1.309	− 0.103
2	DPB1*02:01	DPB1	0.030	1.561	2.659	0.446	2.408	− 0.182	2.040	0.234
3	DPB1*02:02	DPB1	0.117	1.386	2.659	0.654	2.120	− 0.095	1.715	− 0.253
4	DPB1*03:01	DPB1	1.309	1.897	0.041	0.117	0.073	1.273	2.526	0.553
5	DPB1*04:01	DPB1	0.892	0.734	0.892	0.693	0.431	0.416	0.342	− 0.585
6	DPB1*04:02	DPB1	0.357	0.916	1.204	0.139	0.371	− 0.095	1.022	− 0.266
7	DPB1*05:01	DPB1	1.609	2.996	1.966	1.022	1.050	1.347	2.659	0.073
8	DPB1*06:01	DPB1	2.659	1.050	− 0.531	− 0.531	2.526	1.833	1.238	− 0.054
9	DPB1*09:01	DPB1	2.813	0.713	0.020	− 0.693	2.207	2.813	0.916	0.369
10	DPB1*10:01	DPB1	2.526	0.892	− 0.336	− 0.336	2.303	1.897	1.022	0.364
11	DPB1*11:01	DPB1	1.238	1.427	0.528	0.693	1.139	0.654	2.813	− 0.394
12	DPB1*13:01	DPB1	2.996	0.844	0.386	0.030	1.897	1.386	2.659	0.311
13	DPB1*14:01	DPB1	1.204	1.661	0.511	0.371	0.083	1.309	1.661	− 0.102
14	DPB1*17:01	DPB1	2.659	0.654	− 0.531	− 0.262	2.659	1.772	0.916	0.374
15	DPB1*19:01	DPB1	0.400	1.897	2.207	0.073	1.897	0.357	0.511	0.263
16	DQB1*02:01	DQB1	1.273	2.659	1.386	1.238	4.605	0.174	0.868	− 0.117
17	DQB1*02:02	DQB1	1.273	2.659	1.386	1.238	4.605	0.174	0.868	− 0.413
18	DQB1*03:01	DQB1	1.347	0.342	3.912	0.342	3.912	0.545	1.772	0.210
19	DQB1*03:02	DQB1	2.659	3.912	1.470	0.616	4.605	0.916	− 0.095	0.057
20	DQB1*03:03	DQB1	2.303	0.821	3.912	0.128	3.219	0.821	0.105	− 0.050
21	DQB1*04:02	DQB1	2.408	0.616	1.661	− 0.182	3.219	− 0.916	2.408	− 0.080
22	DQB1*05:01	DQB1	3.219	2.040	0.151	− 0.788	2.040	0.371	0.994	− 0.027
23	DQB1*05:02	DQB1	3.507	2.303	− 0.262	− 0.095	1.897	− 0.262	0.163	0.106
24	DQB1*05:03	DQB1	4.605	1.715	0.329	0.062	2.659	− 0.336	1.238	0.260
25	DQB1*06:01	DQB1	1.309	0.868	4.605	0.342	4.605	1.772	− 0.095	0.168
26	DQB1*06:02	DQB1	2.526	0.416	4.605	1.897	2.813	2.303	0.654	− 0.196
27	DQB1*06:03	DQB1	2.526	1.966	2.408	0.654	2.303	− 0.262	0.248	− 0.468
28	DQB1*06:04	DQB1	4.605	3.507	− 0.470	0.371	1.079	0.799	− 0.531	− 0.310
29	DQB1*06:09	DQB1	4.605	4.605	0.030	0.598	3.219	1.139	− 0.470	0.216
30	DRB1*01:01	DRB1	1.661	− 0.095	4.605	− 0.336	1.966	0.174	1.966	− 0.031
31	DRB1*01:02	DRB1	0.211	0.545	0.083	− 1.030	2.303	− 0.336	0.528	− 0.327
32	DRB1*01:03	DRB1	1.561	0.431	3.507	− 1.386	2.526	− 0.916	1.079	− 1.062
33	DRB1*03:01	DRB1	− 0.182	− 0.336	0.478	0.315	0.673	0.446	− 0.642	0.401
34	DRB1*04:01	DRB1	0.580	0.545	1.347	1.109	2.813	0.051	2.408	− 0.319
35	DRB1*04:02	DRB1	1.079	1.514	0.511	0.598	4.605	1.470	3.219	− 0.017
36	DRB1*04:03	DRB1	0.673	0.020	0.386	2.408	0.329	1.171	0.261	− 0.129
37	DRB1*04:04	DRB1	2.040	0.713	1.386	2.303	1.347	2.303	0.799	− 0.017
38	DRB1*04:05	DRB1	0.400	2.207	0.598	0.446	0.400	− 0.993	1.109	0.392
39	DRB1*04:07	DRB1	0.844	1.204	1.309	0.755	1.715	0.094	1.609	− 0.351
40	DRB1*04:08	DRB1	0.713	1.897	1.897	0.693	2.813	1.109	3.219	− 0.166
41	DRB1*07:01	DRB1	2.526	1.772	4.605	− 0.642	2.408	0.446	2.996	− 0.353
42	DRB1*08:01	DRB1	1.139	0.968	1.966	− 0.470	0.186	− 0.405	0.673	− 0.023
43	DRB1*08:03	DRB1	0.635	0.654	1.470	− 0.262	0.128	− 0.588	0.528	0.114
44	DRB1*09:01	DRB1	1.171	1.833	2.659	0.734	4.605	0.000	1.772	− 0.027
45	DRB1*10:01	DRB1	0.580	0.968	4.605	1.609	1.833	1.661	3.912	0.232
46	DRB1*11:01	DRB1	0.462	0.580	2.996	0.128	2.303	− 0.588	2.303	0.406
47	DRB1*11:02	DRB1	0.051	1.715	2.120	0.105	1.561	1.386	2.120	− 0.281
48	DRB1*11:03	DRB1	0.274	0.734	1.470	0.528	2.207	0.693	4.605	0.008
49	DRB1*11:04	DRB1	0.528	1.386	− 0.262	0.821	3.912	0.357	2.813	0.167
50	DRB1*12:01	DRB1	− 0.531	0.942	0.073	− 0.993	3.507	2.303	2.996	− 0.022
51	DRB1*13:01	DRB1	0.051	1.715	2.120	0.105	1.561	1.386	2.120	− 0.123
52	DRB1*13:02	DRB1	0.821	2.408	1.171	1.897	1.966	2.996	1.347	− 0.266
53	DRB1*13:03	DRB1	3.507	1.238	1.139	0.755	0.000	− 0.833	1.514	− 0.373
54	DRB1*13:05	DRB1	0.462	0.580	2.996	0.128	2.303	− 0.588	2.303	− 0.190
55	DRB1*14:01	DRB1	4.605	2.408	− 0.693	− 0.470	4.605	− 0.642	1.966	− 0.016
56	DRB1*15:01	DRB1	3.507	3.219	− 0.833	0.051	3.219	0.041	0.713	− 0.357
57	DRB1*15:02	DRB1	3.219	4.605	2.040	− 0.875	1.204	− 0.833	2.408	− 0.108
58	DRB1*16:01	DRB1	2.207	2.207	3.219	− 0.262	1.897	− 0.262	0.494	0.055

**Figure 1:**
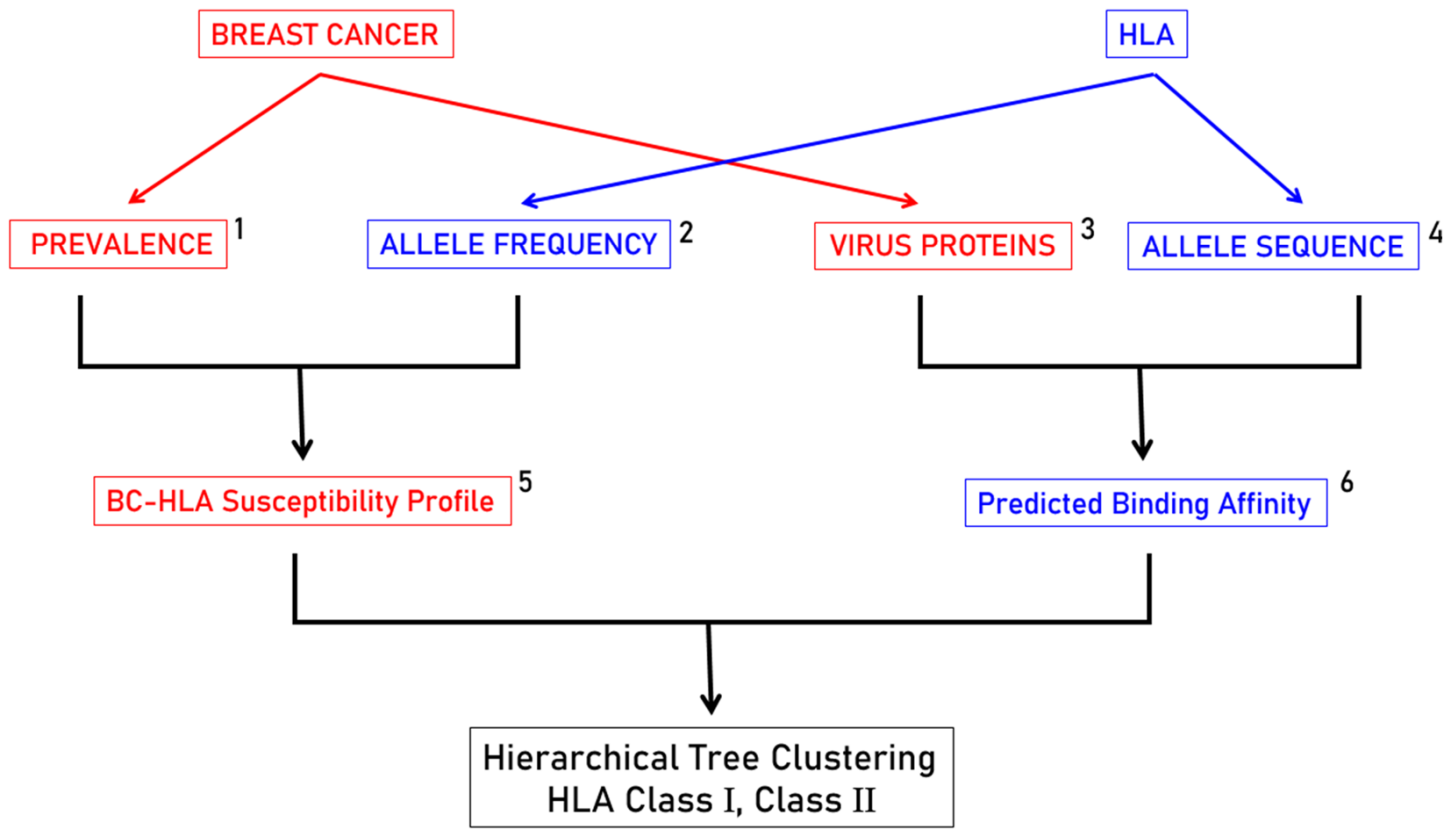
Schematic diagram of the analyses performed. 1, N = 14 prevalences of breast cancer in 14 countries; 2, N = 127 HLA-I and -II alleles; 3, N = 7 virus proteins; 4, N = 127 HLA allele sequences provided by, and used in, the Immune Epitope Database (IEDB) tool^29,30^; 5, N = 127 (BC-HLA P/S score, last column in Tables 2 and 3); 6, N = 127 HLA alleles × 7 viruses = 889 HLA-I (Table 2) and HLA-II (Table 3) estimated binding affinities.

The overall design of our analyses is depicted in the schematic diagram of Fig. 1. Details of the analyses are provided in each of the sections to follow.

### Effect of virus and HLA class on PBA

**Figure 2:**
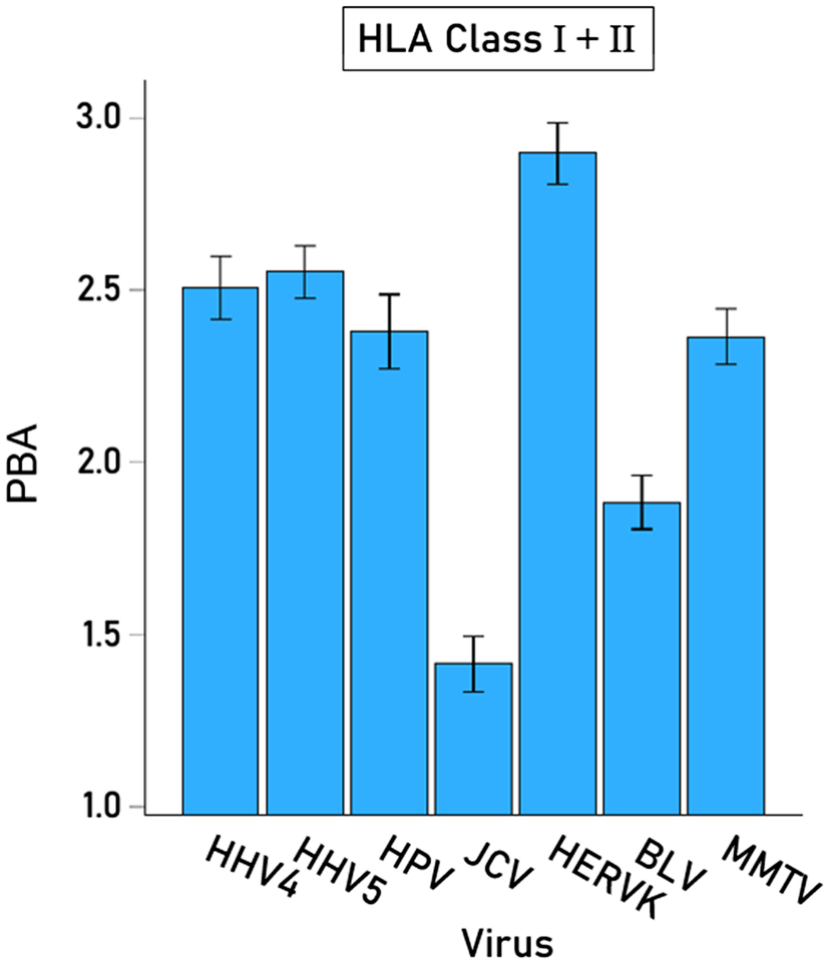
Mean (± SEM) predicted binding affinities of the 7 viral proteins used across all 127 HLA alleles.

**Figure 3:**
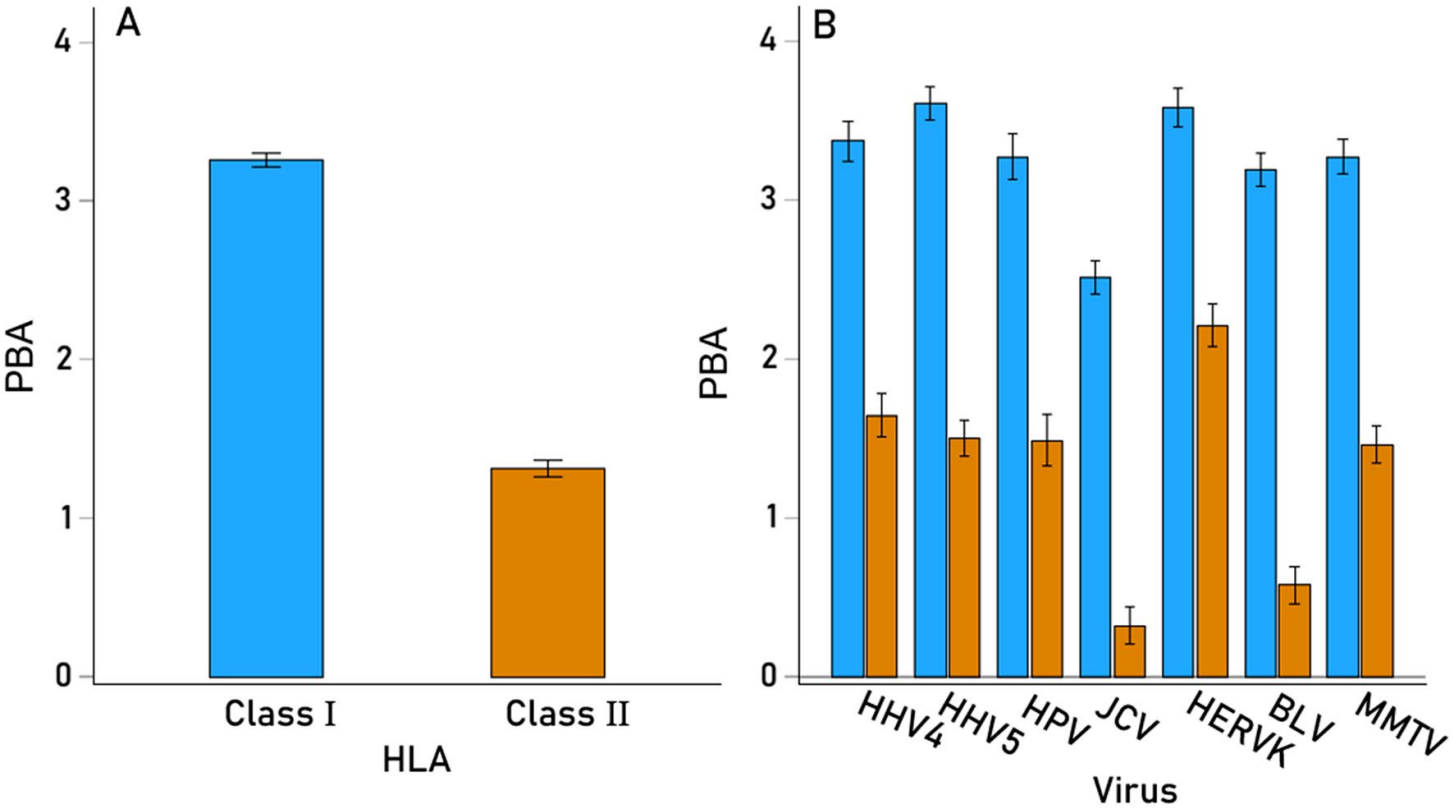
(**A**) Mean (± SEM) predicted binding affinities for HLA-I (N = 69) and HLA-II (N = 58). (**B**) Same for each virus studied.

The effects on PBA of Virus, HLA Class, and their interaction were evaluated using a repeated- measures analysis of variance (ANOVA), where the 7 viruses comprised the “Within-Subjects” Virus factor and the 2 HLA classes comprised the “Between-Subjects” fixed Class factor. We found the following: (a) The effect of Virus was highly significant (P < 0.001, Greenhouse–Geisser test), with JCV and BLV having lower average PBA scores (Fig. 2); (b) The effect of HLA Class was also highly significant (P < 0.001, F-test), with HLA-I having 2.5 × higher scores than HLA-II (Fig. 3A); and (c) the Virus x Class interaction term was also highly significant (P < 0.001, Greenhouse–Geisser test) (Fig. 3B). This interaction seems to be due mainly to the fact that the PBA scores for JCV and BLV viruses are disproportionately lower in HLA-II as compared with HLA-I and are substantially lower than the other viruses.

### Effect of HLA-I and HLA-II genes on PBA

**Figure 4:**
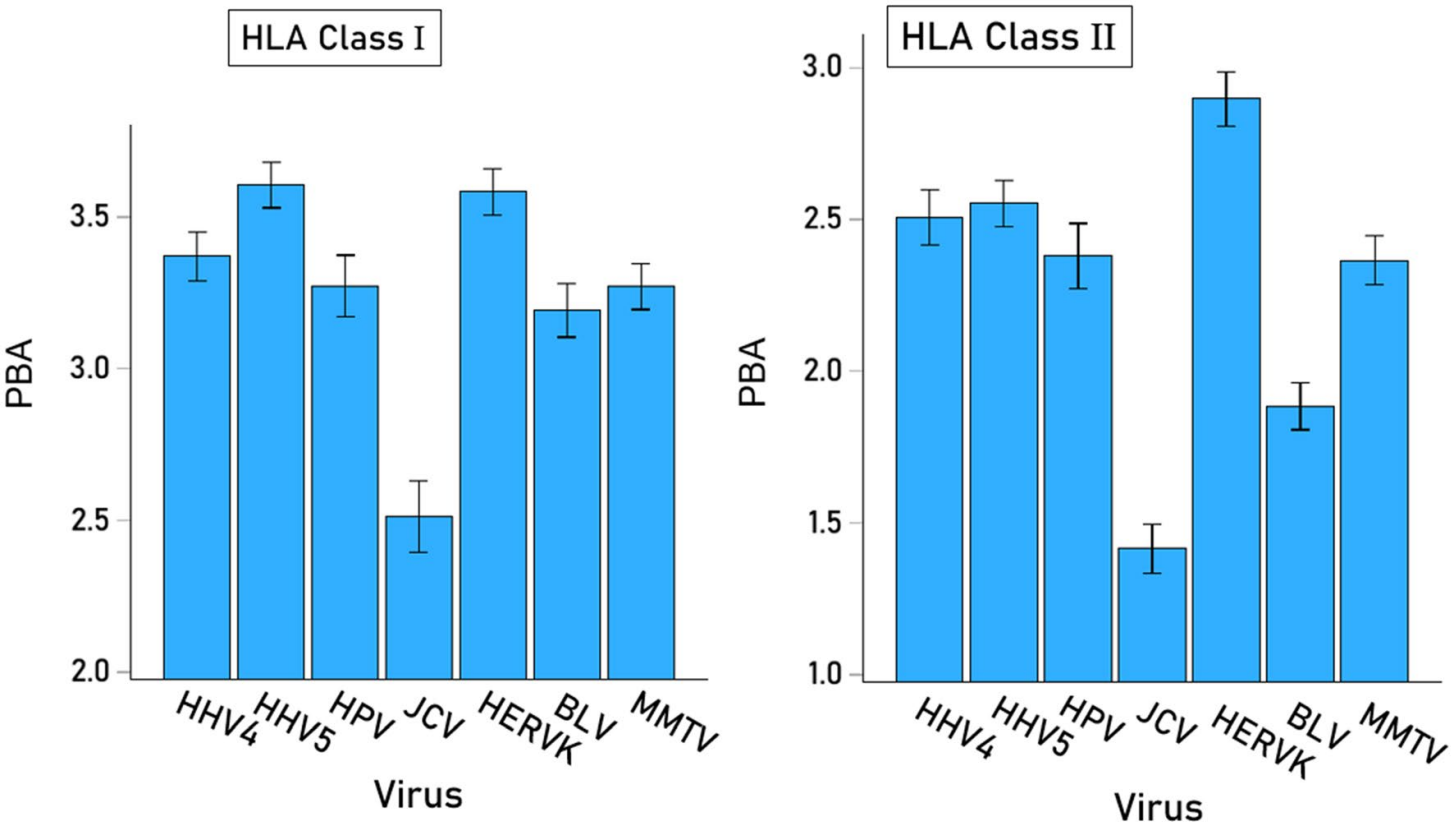
Mean (± SEM) predicted binding affinities for HLA-I and HLA-II for each virus studied.

**Figure 5:**
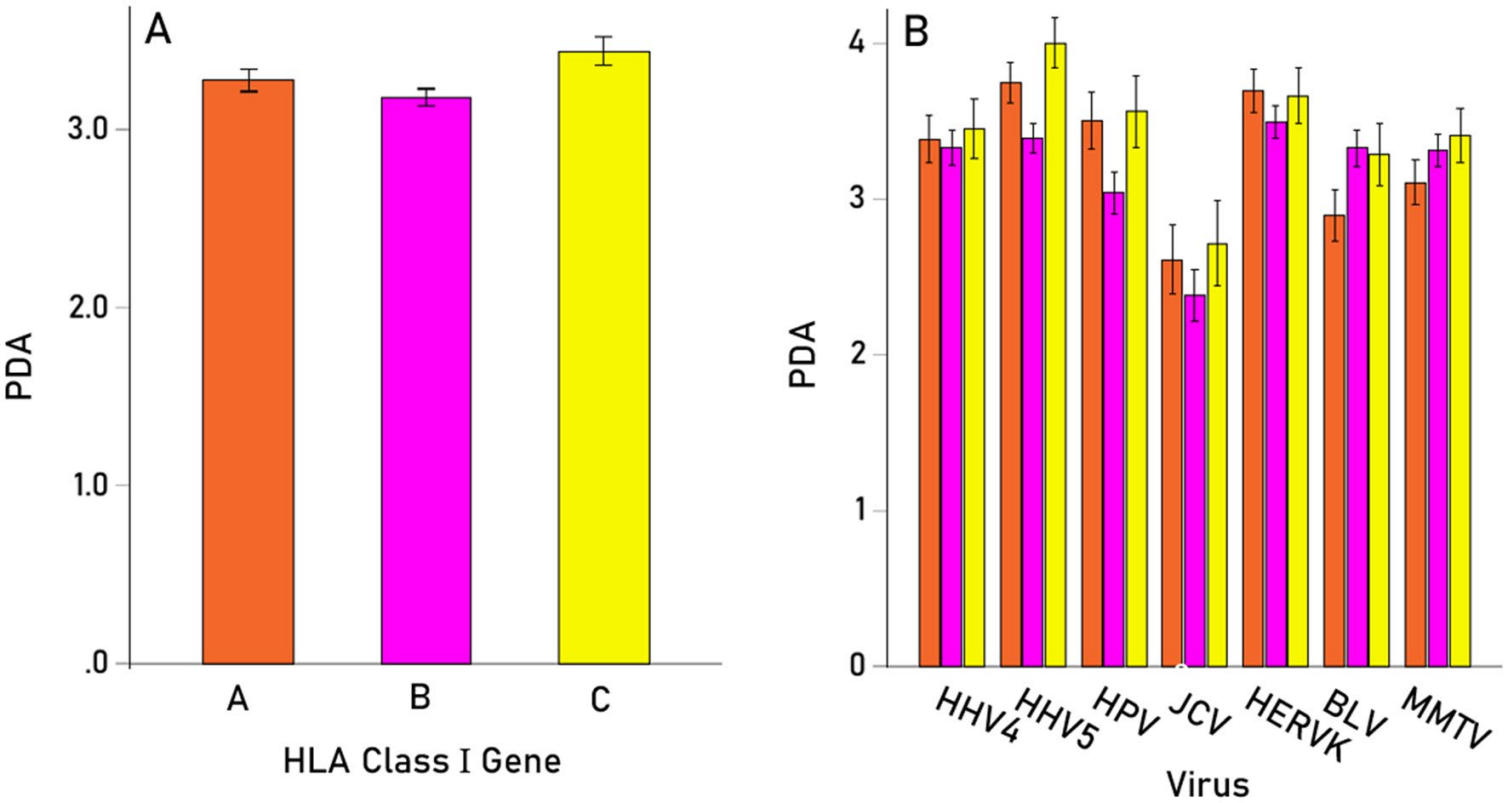
(**A**) Mean (± SEM) predicted binding affinities for HLA-I A, B and C genes (N = 69). (**B**) Same for each virus studied.

**Figure 6:**
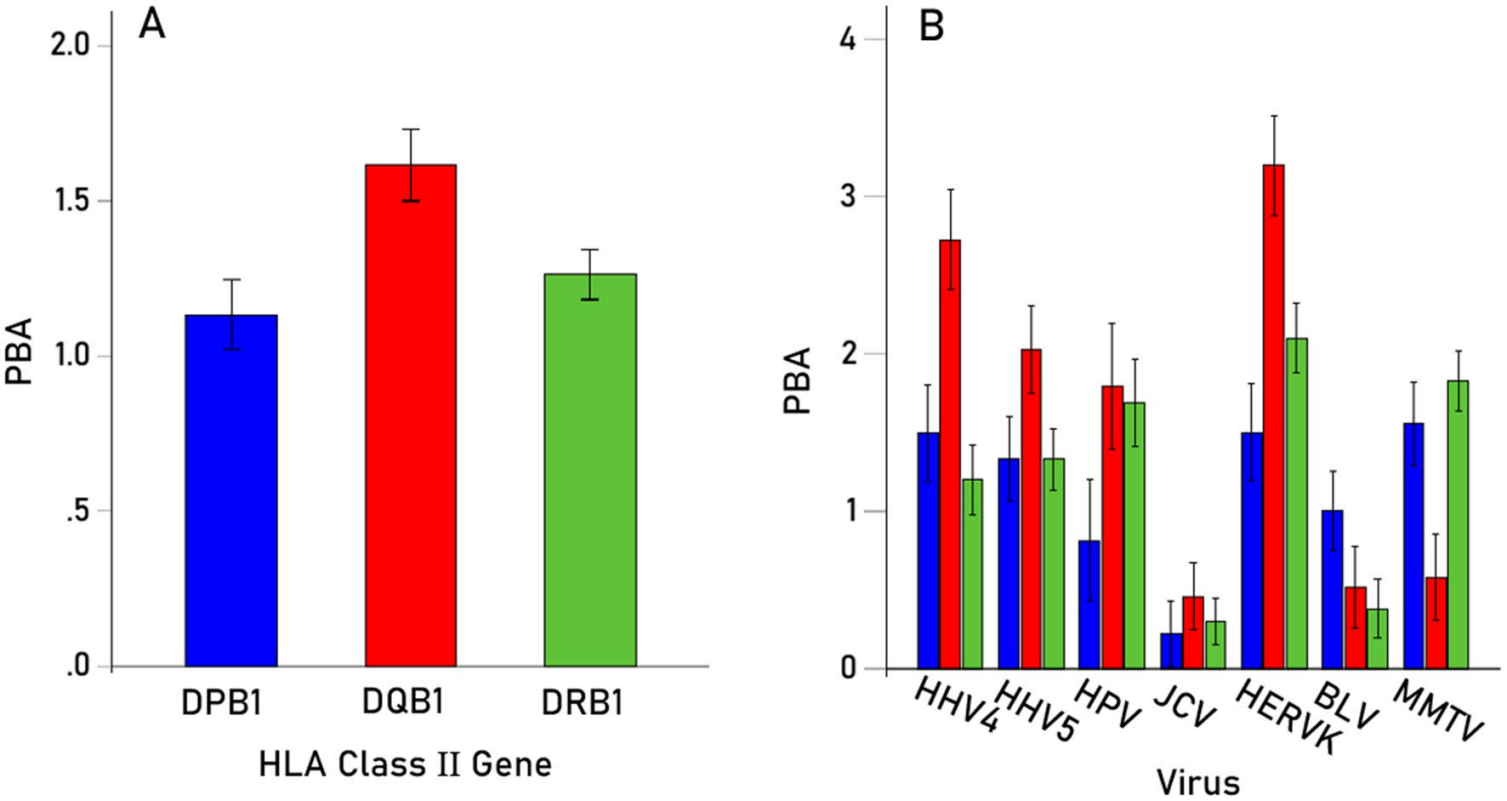
(**A**) Mean (± SEM) predicted binding affinities for HLA-II DPB1, DQB1 and DRB1 genes (N = 58). (**B**) Same for each virus studied.

Given the significant Virus × HLA Class interaction above, the effect of Virus and Gene on PBA were evaluated separately for each HLA class using 2 separate repeated measures ANOVAs, one for each HLA Class, where Virus was the Within-Subjects factor as above, and Gene was the Between-Subjects factor comprising the 3 genes of HLA-I (A, B, C) and the 3 genes of HLA-II (DPB1, DQB1, DRB1). These analyses also evaluated the effect of Virus within each HLA Class separately. We found the following: (a) There was a significant effect of Virus (P < 0.001, Greenhouse–Geisser test) for both HLA-I (Fig. 4, left panel) and HLA-II (Fig. 4, right panel); (b) for HLA-I, there was a marginally significant effect of Gene (P = 0.024, F-test), with higher PBA values for gene C (Fig. 5A), whereas the Virus × Gene effect was not statistically significant (P = 0.166, Greenhouse–Geisser test) (Fig. 5B); (c) For HLA-II, there was a significant effect of Gene (P = 0.011, F-test), with higher PBA values for gene DQB1 (Fig. 6A), and the Virus × Gene effect was highly significant (P < 0.001, Greenhouse–Geisser test) (Fig. 6B).

### Association between PBA and breast cancer: HLA immunogenetic profile

**Figure 7:**
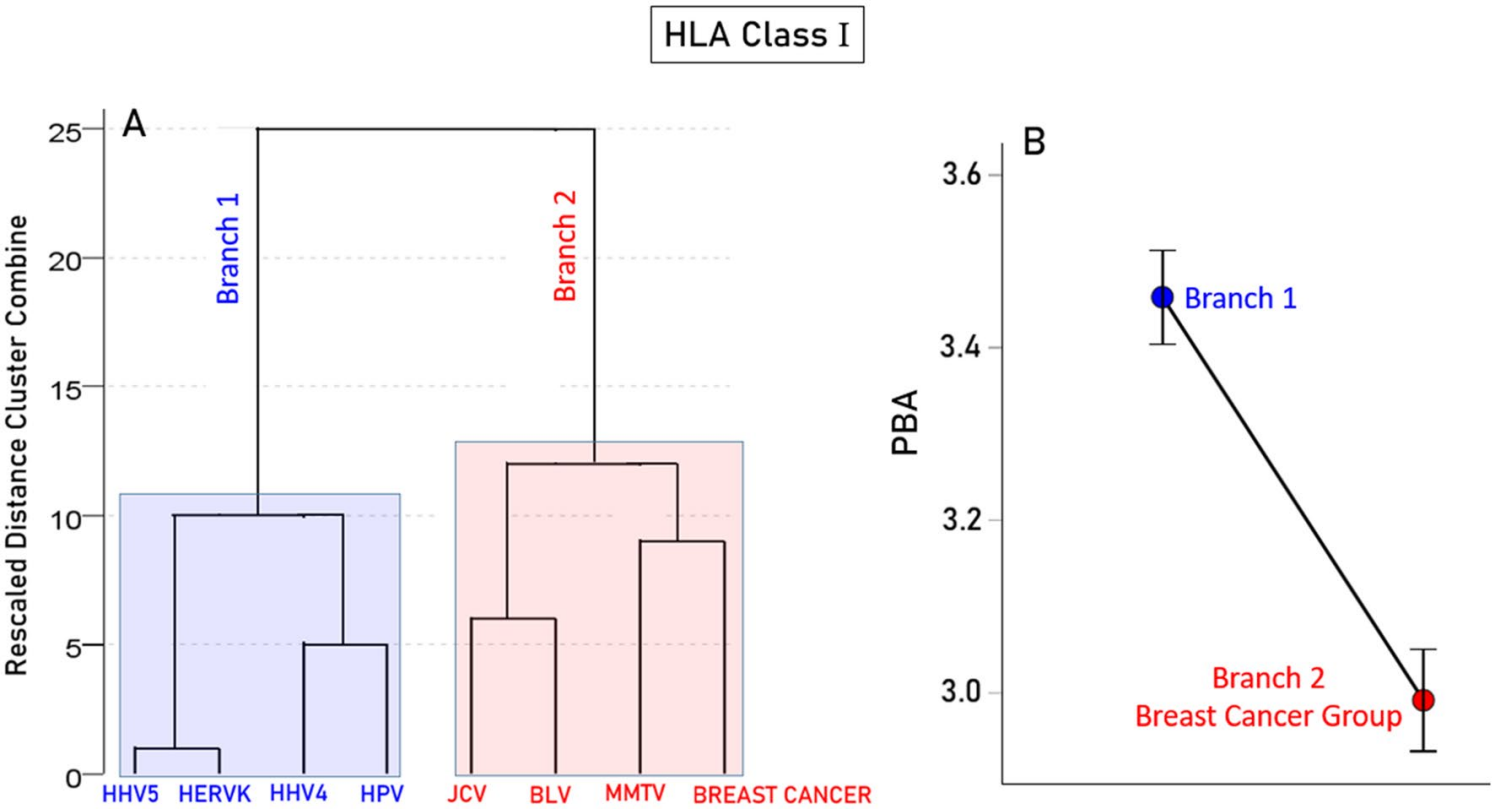
Hierarchical tree clustering results for HLA-I and BC-HLA profile. (**A**) Dendrogram of the 7 viruses’ predicted binding affinities and BC-HLA profile. (**B**) Mean (± SEM) of predicted binding affinities of the viruses in the two color-coded groups of the dendrogram. N = 69 HLA-I alleles. See text for details.

**Figure 8:**
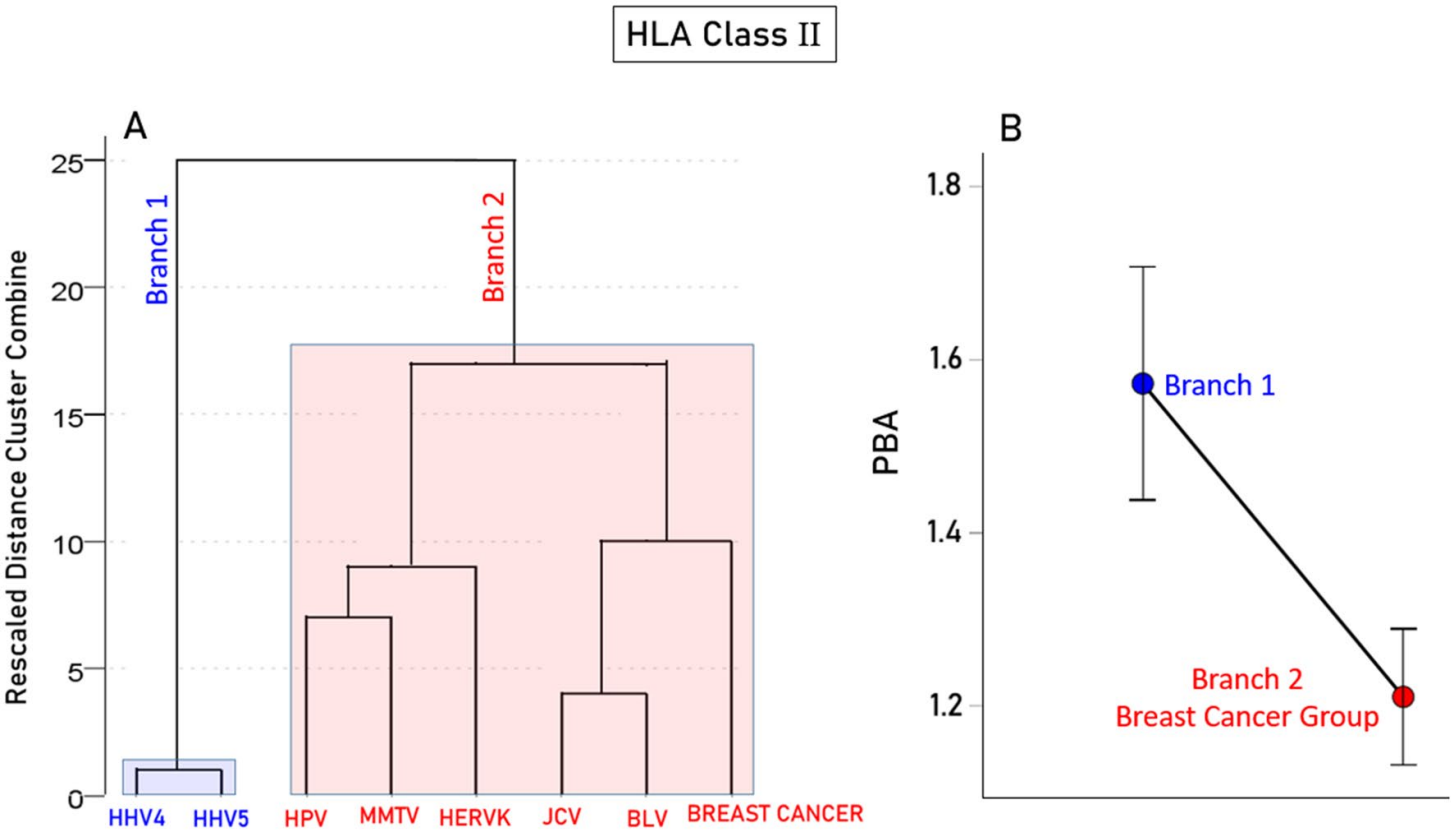
Hierarchical tree clustering results for HLA-II and BC-HLA profile. (**A**) Dendrogram of the 7 viruses’ predicted binding affinities and BC-HLA profile. (**B**) Mean (± SEM) of predicted binding affinities of the viruses in the two color-coded groups of the dendrogram. N = 58 HLA-II alleles. See text for details.

As depicted in the schematic diagram of Fig. 1, our analyses culminated in the hierarchical tree clustering which we applied to the data of HLA-I and HLA-II shown in Tables 2 and 3, respectively. This analysis yielded 2 dendrograms, one for each class. In both cases, there were 2 clusters, as follows. For HLA-I, BC-HLA immunogenetic scores were grouped with BLV, JCV and MMTV (Fig. 7A). Remarkably, the average PBA scores of the viruses in this BC-associated group (red in Fig. 7A,B) were significantly lower than those in the other, non-BC group (blue in Fig. 7A,B) (P < 0.001, paired-sample t-test). For HLA-II, BC-HLA immunogenetic scores were grouped on a sub-branch with JCV and BLV; MMTV, HERV-K, and HPV were grouped on the other sub-branch. As with HLA-I, the average PBA scores of the 5 viruses in the BC-associated group (red in Fig. 8A,B) were significantly lower than those in other non-BC group (P = 0.038, paired samples t-test). Altogether, these results document the grouping of PBA of certain viruses with BC-HLA immunogenetic profile, and their lower predicted binding affinity, as compared to the group of viruses not grouped with BC-HLA.

## Discussion

In light of separate lines of evidence linking both HLA and viruses to breast cancer, we first evaluated the predicted binding affinity of specific viruses implicated in breast cancer with regard to 127 common HLA alleles and then examined the associations between those viral protein binding affinities with a population-derived breast cancer-HLA profile^19^. With regard to the former, the overall results documented variation in HLA-I and HLA-II mediated immunity to viral proteins implicated in breast cancer that varied by virus, HLA gene, and across alleles within each gene. Specifically, our findings documented (a) higher predicting binding affinities of HLA-I alleles (than those of HLA-II), (b) higher binding affinities of gene C of HLA-I and gene DQB1 of HLA-II, and (c) overall lower binding affinities for JCV in both HLA-I and HLA-II.

With respect to viruses, it is worth noting that all 7 viruses investigated here have been implicated in breast cancer^4–9^. This study focused on immunogenetic aspects of these viruses, both with respect to their predicted binding affinities to HLA-I and II alleles and their grouping with breast cancer—HLA immunogenetic profile. A major finding of the latter analysis was the grouping of specific viruses with breast cancer (shaded red in Figs. 7A and 8A), fewer for HLA-I (3/7 viruses, Fig. 7A) than HLA-II (5/7 viruses, Fig. 8A). This grouping enabled us to test the hypothesis that this association of specific viruses with breast cancer immunogenetics may be due to lower virus binding affinity to HLA molecules, thus delaying the elimination of virus directly (via HLA-I—CD8 + engagement leading to death of the infected cell) and/or indirectly (via HLA-II—CD4 + engagement leading to antibody production). Indeed, this was found to be the case for both HLA Classes (Figs. 7B, 8B). It is worth pointing out that the present findings do not preclude possible involvement of viruses in breast cancer via other mechanisms that remain to be identified and investigated.

In summary, the present study provides a novel contribution implicating HLA-mediated virus immunogenicity on breast cancer. Still several limitations must be considered. First, the analyses included 127 common HLA-I and II alleles and 7 viruses. The HLA region, however, is highly polymorphic and the binding affinities of the viruses with other less common HLA alleles was not investigated. Second, although the present analyses focused on 7 viruses that have previously been implicated in breast cancer, other viruses not included here may be involved in breast cancer. Third, we evaluated the binding affinities of HLA molecules to representative proteins of the 7 viruses, all of which are involved with viral entry into the host cell; still, other proteins may have different binding affinities. For example, several hundred types of HPV exist, several of which are associated with high risk for cancer^31^; here, we evaluated HPV16, one of the most common types of HPV involved in cancer risk^32^, yet other types of HPV may have different binding affinities. Finally, the breast cancer-HLA profile was based on population prevalence of breast cancer in general^19^; specific types of breast cancer may have a different HLA profile. Despite these limitations, the current findings provide novel insights regarding the interaction of virus exposure and host immunogenetics with regard to breast cancer.

## Materials and methods

### HLA alleles

We obtained the population frequency in 2019 of 69 common HLA-I alleles and 58 common HLA-II alleles that occurred in at least 9 of 14 Continental Western European Countries (Austria, Belgium, Denmark, Finland, France, Germany, Greece, Italy, Netherlands, Portugal, Norway, Spain, Sweden, and Switzerland) at frequencies ≥ 0.01, as described previously^33^.

### Breast cancer: HLA protection/susceptibility (P/S) scores

These scores are correlations (Fisher z-transformed) between the prevalence of breast cancer in the 14 countries above and the population frequency of each one of the 127 HLA alleles in the same countries. The scores have been published^19^ and are given in Tables 2 and 3.

### Virus proteins

We estimated in silico the predicted binding affinities (for each one of the 127 HLA alleles) of proteins of 7 viruses that have been implicated in breast cancer, namely HHV4, HHV5, HPV, JCV, HERVK, BLV, and MMTV. Details of the proteins analyzed are given in Table 1 and their amino acid (AA) sequences are given in the Appendix (Supplementary Materials).

### In silico determination of predicted binding affinity of HLA-I and HLA-II alleles

**Figure 9:**
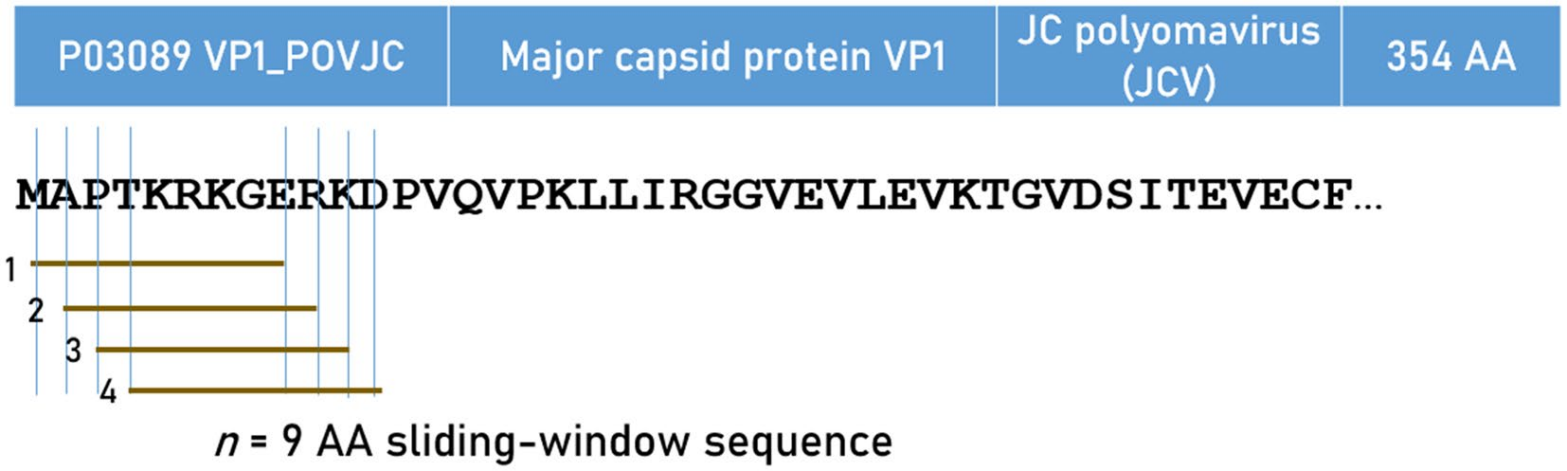
Illustration of the 9-mer sliding window approach for the in silico estimation of the predicted binding activity for HLA-I alleles.

**Figure 10:**
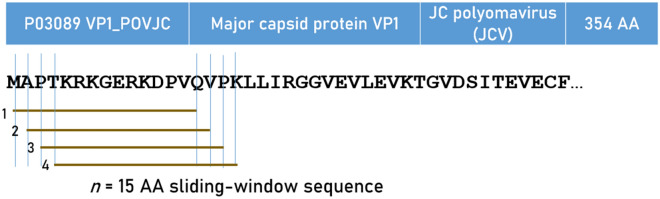
Illustration of the 15-mer sliding window approach for the in silico estimation of the predicted binding activity for HLA-II alleles.

Predicted binding affinities were obtained for viral protein epitopes using the Immune Epitope Database (IEDB) NetMHCpan (ver. 4.1) tool^29,30^. More specifically, we used the sliding window approach^34–36^ to test exhaustively all possible linear 9-mer (for HLA-I predictions) and 15-mer (for HLA-II predictions) AA residue epitopes of the 7 viral proteins analyzed (Table 1). The method is illustrated in Figs. 9 and 10 for the JCV virus protein. For each epitope-HLA molecule tested, this tool gives, as an output, the percentile rank of binding affinity of the HLA molecule and the epitope among predicted binding affinities of the same HLA molecule to a large number of different peptides of the same AA length; the smaller the percentile rank, the better the binding affinity. Now, given a protein of *N* amino acid length and an epitope length of *k* AA, there are *N–k* binding affinity predictions, i.e. *N–k* percentile ranks. Of these predictions, for each viral protein and HLA molecule tested, we retained the lowest percentile rank (LPR) as the best possible binding affinity of the protein-HLA molecule pair. We then applied two transformations on LPR. First, we took its inverse, so that higher values mean better binding affinities for more intuitive interpretation:

1 $$LP{R}^{\prime}=\frac{1}{LPR}$$

**Figure 11:**
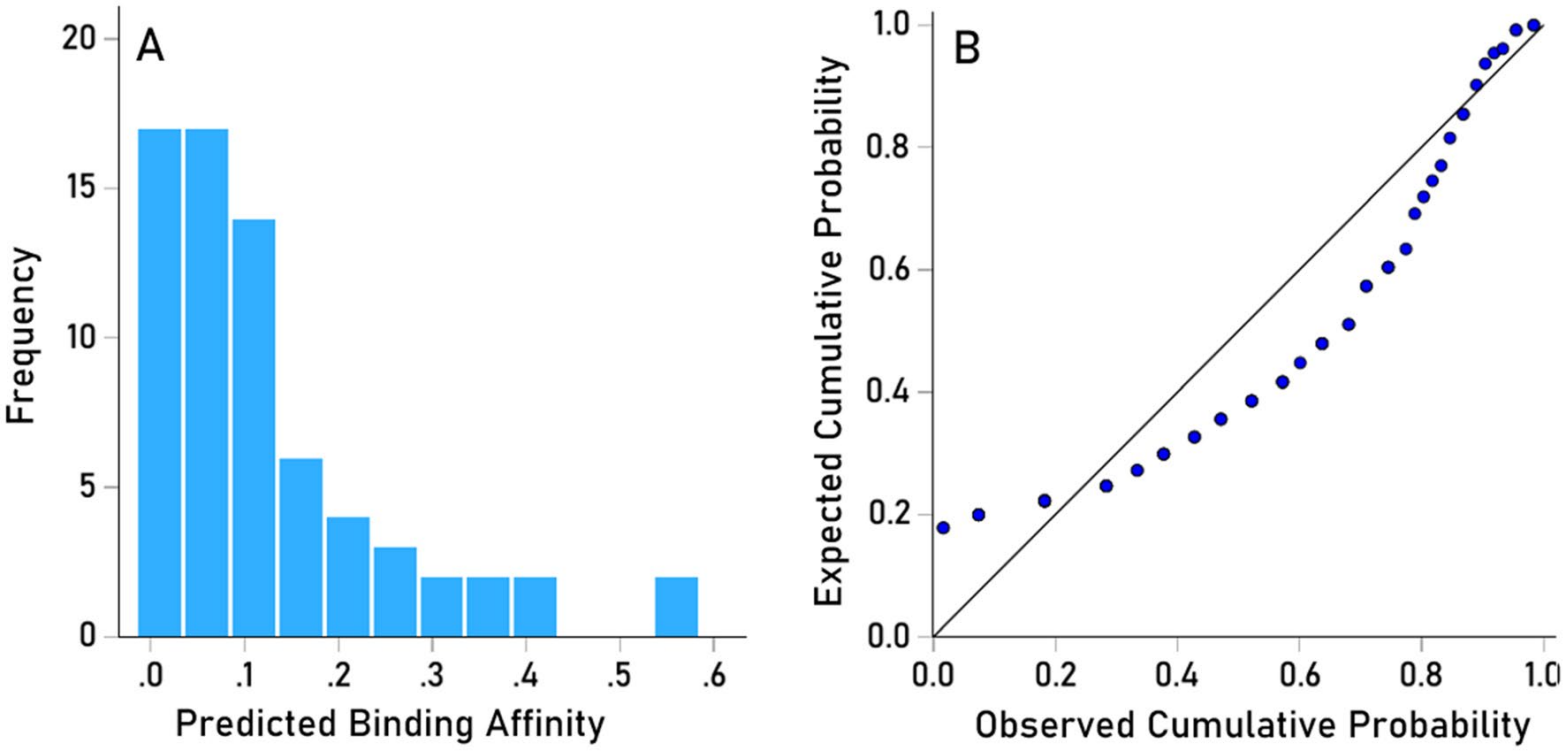
(**A**) frequency distribution of raw (untransformed) predicted binding affinities ($LP{R}^{\prime})$ to illustrate their deviation from a symmetric distribution. (**B**) Probability-probability plot of the data in (**A**). Data from JCV virus.

**Figure 12:**
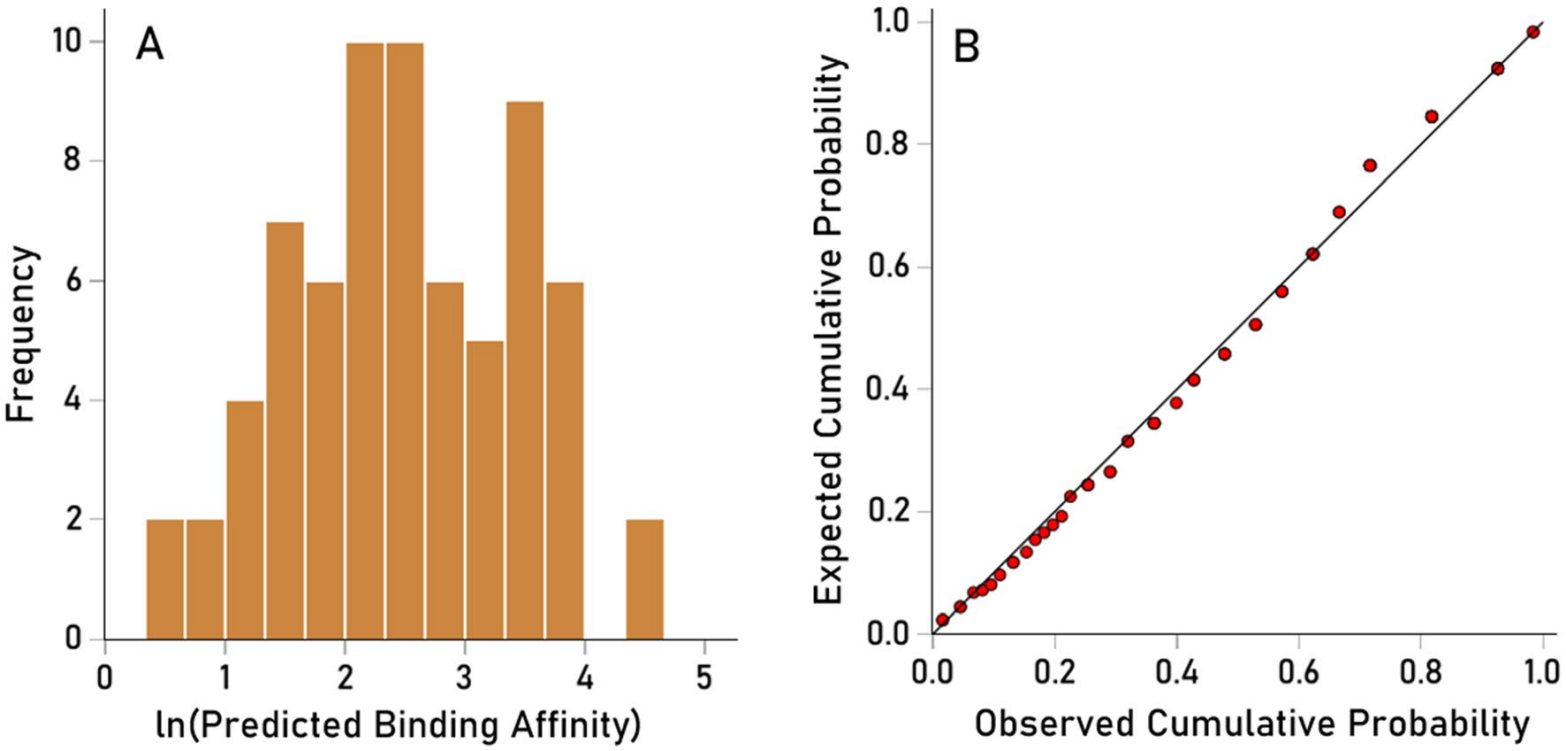
(**A**) frequency distribution of raw (untransformed) predicted binding affinities ($LP{R}^{\prime})$ to illustrate their deviation from a symmetric distribution. (**B**) Probability-probability plot of the data in (**A**). Data from JCV virus.

The $LPR^{\prime}$ distribution was heavily skewed to the left (Fig. 11A), resembling a exponential distribution and deviating substantially from a normal distribution (Fig. 11B). Therefore, $LPR^{\prime}$ values were (natural) log transformed to normalize the distribution for quantitative analyses (Fig. 12A,B):

2 $$\text{Predicted Binding Affinity }(\text{PBA}): PBA=\text{ln}(LP{R}^{\prime})$$

Give the logarithmic transformation above, PBA > 0 indicate $LP{R}^{\prime}>1$, whereas PBA < 0 indicate $LP{R}^{\prime}<1$.

### Statistical analyses

#### General

Standard statistical methods were used to analyze the data using the IBM-SPSS statistical package (version 29), including analysis of variance (ANOVA), t-test, etc. All P-values reported are 2-sided. In addition, hierarchical tree clustering was performed on standardized data (Z-scores), with Ward linkage as the method and squared Euclidean distance as the interval.

### Ethical approval

This article does not contain any studies with human participants performed by any of the authors.

### Supplementary Information

Supplementary Information.

## Data Availability

All data used were retrieved from freely accessible websites [Ref.^29^: http://allelefrequencies.net/hla6006a.asp; Ref.^31^: http://tools.iedb.org/mhci/result/ or open access publication [Ref.^17^10.1177/11769351221148588] and, as such, are publicly and freely available.
