# Supplementary material for: Breast cancer, viruses, and human leukocyte antigen (HLA)
Source: Sci Rep. 2024 Jul 13;14:16179. doi: 10.1038/s41598-024-65707-9 (PMC11246526; doi:10.1038/s41598-024-65707-9)
Supplement: Supplementary file 1 — Supplementary Information. [file 41598_2024_65707_MOESM1_ESM.docx]

**APPENDIX**

Amino acid sequences of the 7 viruses analyzed (Table 1). Labels are from Uniprot (<https://www.uniprot.org/uniprotkb/>).

| Human herpes virus 4 P03188 | Envelope glycoprotein B | 857 AA |
| --- | --- | --- |

MTRRRVLSVVVLLAALACRLGAQTPEQPAPPATTVQPTATRQQTSFPFRVCELSSHGDLFRFSSDIQCPSFGTRENHTEGLLMVFKDNIIPYSFKVRSYTKIVTNILIYNGWYADSVTNRHEEKFSVDSYETDQMDTIYQCYNAVKMTKDGLTRVYVDRDGVNITVNLKPTGGLANGVRRYASQTELYDAPGWLIWTYRTRTTVNCLITDMMAKSNSPFDFFVTTTGQTVEMSPFYDGKNKETFHERADSFHVRTNYKIVDYDNRGTNPQGERRAFLDKGTYTLSWKLENRTAYCPLQHWQTFDSTIATETGKSIHFVTDEGTSSFVTNTTVGIELPDAFKCIEEQVNKTMHEKYEAVQDRYTKGQEAITYFITSGGLLLAWLPLTPRSLATVKNLTELTTPTSSPPSSPSPPAPSAARGSTPAAVLRRRRRDAGNATTPVPPTAPGKSLGTLNNPATVQIQFAYDSLRRQINRMLGDLARAWCLEQKRQNMVLRELTKINPTTVMSSIYGKAVAAKRLGDVISVSQCVPVNQATVTLRKSMRVPGSETMCYSRPLVSFSFINDTKTYEGQLGTDNEIFLTKKMTEVCQATSQYYFQSGNEIHVYNDYHHFKTIELDGIATLQTFISLNTSLIENIDFASLELYSRDEQRASNVFDLEGIFREYNFQAQNIAGLRKDLDNAVSNGRNQFVDGLGELMDSLGSVGQSITNLVSTVGGLFSSLVSGFISFFKNPFGGMLILVLVAGVVILVISLTRRTRQMSQQPVQMLYPGIDELAQQHASGEGPGINPISKTELQAIMLALHEQNQEQKRAAQRAAGPSVASRALQAARDRFPGLRRRRYHDPETAAALLGEAETEF

| HHV5: P06473 · GB_HCMVA | Envelope glycoprotein B | gB | 906 AA |
| --- | --- | --- | --- |

MESRIWCLVVCVNLCIVCLGAAVSSSSTSHATSSTHNGSHTSRTTSAQTRSVYSQHVTSSEAVSHRANETIYNTTLKYGDVVGVNTTKYPYRVCSMAQGTDLIRFERNIICTSMKPINEDLDEGIMVVYKRNIVAHTFKVRVYQKVLTFRRSYAYIYTTYLLGSNTEYVAPPMWEIHHINKFAQCYSSYSRVIGGTVFVAYHRDSYENKTMQLIPDDYSNTHSTRYVTVKDQWHSRGSTWLYRETCNLNCMLTITTARSKYPYHFFATSTGDVVYISPFYNGTNRNASYFGENADKFFIFPNYTIVSDFGRPNAAPETHRLVAFLERADSVISWDIQDEKNVTCQLTFWEASERTIRSEAEDSYHFSSAKMTATFLSKKQEVNMSDSALDCVRDEAINKLQQIFNTSYNQTYEKYGNVSVFETSGGLVVFWQGIKQKSLVELERLANRSSLNITHRTRRSTSDNNTTHLSSMESVHNLVYAQLQFTYDTLRGYINRALAQIAEAWCVDQRRTLEVFKELSKINPSAILSAIYNKPIAARFMGDVLGLASCVTINQTSVKVLRDMNVKESPGRCYSRPVVIFNFANSSYVQYGQLGEDNEILLGNHRTEECQLPSLKIFIAGNSAYEYVDYLFKRMIDLSSISTVDSMIALDIDPLENTDFRVLELYSQKELRSSNVFDLEEIMREFNSYKQRVKYVEDKVVDPLPPYLKGLDDLMSGLGAAGKAVGVAIGAVGGAVASVVEGVATFLKNPFGAFTIILVAIAVVIITYLIYTRQRRLCTQPLQNLFPYLVSADGTTVTSGSTKDTSLQAPPSYEESVYNSGRKGPGPPSSDASTAAPPYTNEQAYQMLLALARLDAEQRAQQNGTDSLDGQTGTQDKGQKPNLLDRLRHRKNGYRHLKDSDEEENV

| JC polyomavirus (JCV)  P03089 VP1_POVJC | Major capsid protein VP1 | 354 AA |
| --- | --- | --- |

MAPTKRKGERKDPVQVPKLLIRGGVEVLEVKTGVDSITEVECFLTPEMGDPDEHLRGFSKSISISDTFESDSPNRDMLPCYSVARIPLPNLNEDLTCGNILMWEAVTLKTEVIGVTSLMNVHSNGQATHDNGAGKPVQGTSFHFFSVGGEALELQGVLFNYRTKYPDGTIFPKNATVQSQVMNTEHKAYLDKNKAYPVECWVPDPTRNENTRYFGTLTGGENVPPVLHITNTATTVLLDEFGVGPLCKGDNLYLSAVDVCGMFTNRSGSQQWRGLSRYFKVQLRKRRVKNPYPISFLLTDLINRRTPRVDGQPMYGMDAQVEEVRVFEGTEELPGDPDMMRYVDKYGQLQTKML

| Human papillomavirus Q81007 | Major capsid protein L1 | 494 AA |
| --- | --- | --- |

TVYLPPVPVSKVVSTDEYVARTNIYYHAGTSRLLAVGHPYFPIKKPNNNKILVPKVSGLQYRVFRIHLPDPNKFGFPDTSFYNPDTQRLVWACVGVEVGRGQPLGVGISGHPLLNKLDDTENASAYAANAGVDNRECISMDYKQTQLCLIGCKPPIGEHWGKGSPCTNVAVNPGDCPPLELINTVIQDGDMVDTGFGAMDFTTLQANKSEVPLDICTSICKYPDYIKMVSEPYGDSLFFYLRREQMFVRHLFNRAGTVGENVPDDLYIKGSGSTANLASSNYFPTPSGSMVTSDAQIFNKPYWLQRAQGHNNGICWGNQLFVTVVDTTRSTNMSLCAAISTSETTYKNTNFKEYLRHGEEYDLQFIFQLCKITLTADVMTYIHSMNSTILEDWNFGLQPPPGGTLEDTYRFVTSQAIACQKHTPPAPKEDPLKKYTFWEVNLKEKFSADLDQFPLGRKFLLQAGLKAKPKFTLGKRKATPTTSSTSTTAKRKKR

| Mouse mammary tumor virus P03374 | Envelope glycoprotein gp70 | 688 AA |
| --- | --- | --- |

MPNHQSGSPTGSSDLLLSGKKQRPHLALRRKRRREMRKINRKVRRMNLAPIKEKTAWQHLQALISEAEEVLKTSQTPQNSLTLFLALLSVLGPPPVTGESYWAYLPKPPILHPVGWGSTDPIRVLTNQTMYLGGSPDFHGFRNMSGNVHFEGKSDTLPICFSFSFSTPTGCFQVDKQVFLSDTPTVDNNKPGGKGDKRRMWELWLHTLGNSGANTKLVPIKKKLPPKYPHCQIAFKKDAFWEGDESAPPRWLPCAFPDKGVSFSPKGALGLLWDFSLPSPSVDQSDQIKSKKDLFGNYTPPVNKEVHRWYEAGWVEPTWFWENSPKDPNDRDFTALVPHTELFRLVAASRHLILKRPGFQEHEMIPTSACVTYPYAILLGLPQLIDIEKRGSTFHISCSSCRLTNCLDSSAYDYAAIIVKRPPYVLLPVDIGDEPWFDDSAIQTFRYATDLIRAKRFVAAIILGISALIAIITSFAVATTALVKEMQTATFVNNLHRNVTLALSEQRIIDLKLEARLNALEEVVLELGQDVANLKTRMSTRCHANYDFICVTPLPYNATEDWERTRAHLLGIWNDNEISYNIQELTNLISDMSKQHIDAVDLSGLAQSFANGVKALNPLDWTQYFIFIGVGALLLVIVLMIFPIVFQCLAKSLDQVQSDLNVLLLKKKKGGNAAPAAEMVELPRVSYT

| Bovine leukemia virus P51519 | Envelope glycoprotein | 515 AA |
| --- | --- | --- |

MPKERRSRRRPQPIIRWVSLTLTLLALCQPIQTWRCSLSLGNQQWMTTYNQEAKFSISIDQILEAHNQSPFCPRSPRYTLDFVNGYPKIYWPPPQGRRRFGARAMVTYDCEPRCPYVGADHFDCPHWDNASQADQGSFYVNHQILFLHLKQCHGIFTLTWEIWGYDPLITFSLHKIPDPPQPDFPQLNSDWVPSVRSWALLLNQTARAFPDCAICWEPSPPWAPEILVYNKTISGSGPGLALPDAQIFWVNTSLFNTTQGWHHPSQRLLFNVSQGNALLLPPISLVNLSTVSSAPPTRVRRSPVAALTLGLALSVGLTGINVAVSALSHQRLTSLIHVLEQDQQRLITAINQTHYNLLNVASVVAQNRRGLDWLYIRLGFQSLCPTINEPCCFLRIQNDSIIRLGDLQPLSQRVSTDWQWPWNWDLGLTAWVRETIHSVLSLFLLALFLLFLAPCLIKCLTSRLLKLLRQAPHFPEISFPPKPDSDYQALLPSAPEIYSHLSPTKPDYINLRPCP

| Human endogenous retrovirus group K P10266 | Endogenous retrovirus group K member 10 Pol protein | 1014 AA |
| --- | --- | --- |

NKSRKRRNRVSFLGAVTVEPPKPIPLTWKTEKPVWVNQWPLPKQKLEALHLLANEQLEKGHIEPSFSPWNSPVFVIQKKSGKWHTLTDLRAVNAVIQPMGPLQPGLPSPAMIPKDWPLIIIDLKDCFFTIPLAEQDCEKFAFTIPAINNKEPATRFQWKVLPQGMLNSPTICQTFVGRALQPVREKFSDCYIIHYIDDILCAAETKDKLIDCYTFLQAEVANAGLAIASDKIQTSTPFHYLGMQIENRKIKPQKIEIRKDTLKTLNDFQKLLGDINWIRPTLGIPTYAMSNLFSILRGDSDLNSQRILTPEATKEIKLVEEKIQSAQINRIDPLAPLQLLIFATAHSPTGIIIQNTDLVEWSFLPHSTVKTFTLYLDQIATLIGQTRLRITKLCGNDPDKIVVPLTKEQVRQAFINSGAWQIGLANFVGLIDNHYPKTKIFQFLKLTTWILPKITRREPLENALTVFTDGSSNGKAAYTGPKERVIKTPYQSAQRDELVAVITVLQDFDQPINIISDSAYVVQATRDVETALIKYSMDDQLNQLFNLLQQTVRKRNFPFYITYIRAHTNLPGPLTKANEQADLLVSSALIKAQELHALTHVNAAGLKNKFDVTWKQAKDIVQHCTQCQVLHLPTQEAGVNPRGLCPNALWQMDVTHVPSFGRLSYVHVTVDTYSHFIWATCQTGESTSHVKKHLLSCFAVMGVPEKIKTDNGPGYCSKAFQKFLSQWKISHTTGIPYNSQGQAIVERTNRTLKTQLVKQKEGGDSKECTTPQMQLNLALYTLNFLNIYRNQTTTSAEQHLTGKKNSPHEGKLIWWKDNKNKTWEIGKVITWGRGFACVSPGENQLPVWLPTRHLKFYNEPIGDAKKRASTEMVTPVTWMDNPIEVYVNDSIWVPGPIDDRCPAKPEEEGMMINISIGYRYPPICLGRAPGCLMPAVQNWLVEVPTVSPISRFTYHMVSGMSLRPRVNYLQDFSYQRSLKFRPKGKPCPKEIPKESKNTEVLVWEECVANSAVIL
